# GATA3 recruits UTX for gene transcriptional activation to suppress metastasis of breast cancer

**DOI:** 10.1038/s41419-019-2062-7

**Published:** 2019-11-04

**Authors:** Wenqian Yu, Wei Huang, Yang Yang, Rongfang Qiu, Yi Zeng, Yongqiang Hou, Gancheng Sun, Hang Shi, Shuai Leng, Dandan Feng, Yang Chen, Shuang Wang, Xu Teng, Hefen Yu, Yan Wang

**Affiliations:** 1 2011 Collaborative Innovation Center of Tianjin for Medical Epigenetics, Tianjin Key Laboratory of Medical Epigenetics, Key Laboratory of Immune Microenvironment and Disease (Ministry of Education), Department of Biochemistry and Molecular Biology, School of Basic Medical Sciences, Tianjin Medical University, 300070 Tianjin, P.R. China; 2 Cardiovascular surgery center, Shandong Provincial ENT Hospital affiliated to Shandong University, 250022 Jinan, P.R. China; 3 Beijing Key Laboratory for Tumor Invasion and Metastasis, Advanced Innovation Center for Human Brain Protection, Department of Biochemistry and Molecular Biology, School of Basic Medical Sciences, Capital Medical University, 100069 Beijing, P.R. China

**Keywords:** Breast cancer, Metastasis, Tumour-suppressor proteins

## Abstract

GATA3 has emerged as a prominent transcription factor required for maintaining mammary-gland homeostasis. GATA3 loss is associated with aggressive breast cancer development, but the mechanism by which breast cancer is affected by the loss of GATA3 function remains unclear. Here, we report that GATA3 expression is positively correlated with the expression of UTX, a histone H3K27 demethylase contained in the MLL4 methyltransferase complex, and that GATA3 recruits the chromatin-remodeling MLL4 complex and interacts directly with UTX, ASH2L, and RBBP5. Using RNA sequencing and chromatin immunoprecipitation and sequencing, we demonstrate that the GATA3/UTX complex synergistically regulates a cohort of genes including *Dicer* and *UTX*, which are critically involved in the epithelial-to-mesenchymal transition (EMT). Our results further show that the GATA3-UTX-Dicer axis inhibits EMT, invasion, and metastasis of breast cancer cells in vitro and the dissemination of breast cancer in vivo. Our study implicates the GATA3-UTX-Dicer axis in breast cancer metastasis and provides new mechanistic insights into the pathophysiological function of GATA3.

## Introduction

GATA transcription factors play a crucial role in the gene regulatory networks that control cell-fate specification^1,2^. The GATA family comprises 6 highly conserved transcription factors (GATA1-6) that bind to a functionally important DNA sequence (A/T)GATA(A/G), and GATA proteins are involved in diverse cellular processes, including cell proliferation, differentiation, metabolism, DNA repair, and senescence^3–5^. GATA3 was reported to be required for maintaining luminal epithelial-cell differentiation^6,7^, with GATA3 being expressed exclusively in the luminal cells and absent in myoepithelial cells. As a cell-differentiation regulator, GATA3 is a strong and independent predictor of tumor grade, estrogen-receptor (ER) status, and clinical outcome in human breast cancer^8^. In both animal and human cells, GATA3 acts as a tumor suppressor by directly inhibiting epithelial-to-mesenchymal transition (EMT)^9,10^. Although dysregulation of GATA3 expression in breast cancer has been widely reported in previous studies, the mechanistic involvement of GATA3 in breast cancer dissemination and metastasis remains to be investigated, and how GATA3 functions in the development and progression of breast cancer is currently poorly understood.

Epigenetic regulation, which involves enzymatic modification of histones without disturbing DNA sequences, plays a crucial role in controlling gene expression to orchestrate distinct biological processes^11,12^. UTX, the ubiquitously transcribed X-chromosome tetratricopeptide-repeat protein (also named KDM6A), is a histone demethylase that demethylates both di- and tri-methylated Lys27 on histone H3 (H3K27me2/H3K27me3)^13^. UTX is part of a transcriptional activator complex that includes the MLL3/MLL4 H3K4 methyltransferases, which are linked to homeotic gene expression, cellular reprogramming, embryonic development, as well to as tumor suppression^14–16^. Recent high-throughput genome-wide analyses characterizing the mutational landscape of multiple cancer types have identified inactivating mutations/deletions in UTX that frequently occur in breast cancer, renal cancer, bladder cancer, and leukemia^17–19^. However, the role of UTX as an oncogene or a tumor suppressor in breast cancer remains debated, and its mechanism of action underlying breast cancer progression requires further elucidation.

In this study, we found that GATA3 recruits UTX for gene transcriptional activation. We analyzed the genomic targets and potential function involved in breast cancer progression of this protein. Our findings identify a previously unrecognized mechanism for driving tumor suppression in mammary epithelial cells.

## Results

### In silico analysis of breast cancer databases reveals correlation between expression of GATA3 and UTX

**Fig. 1:**
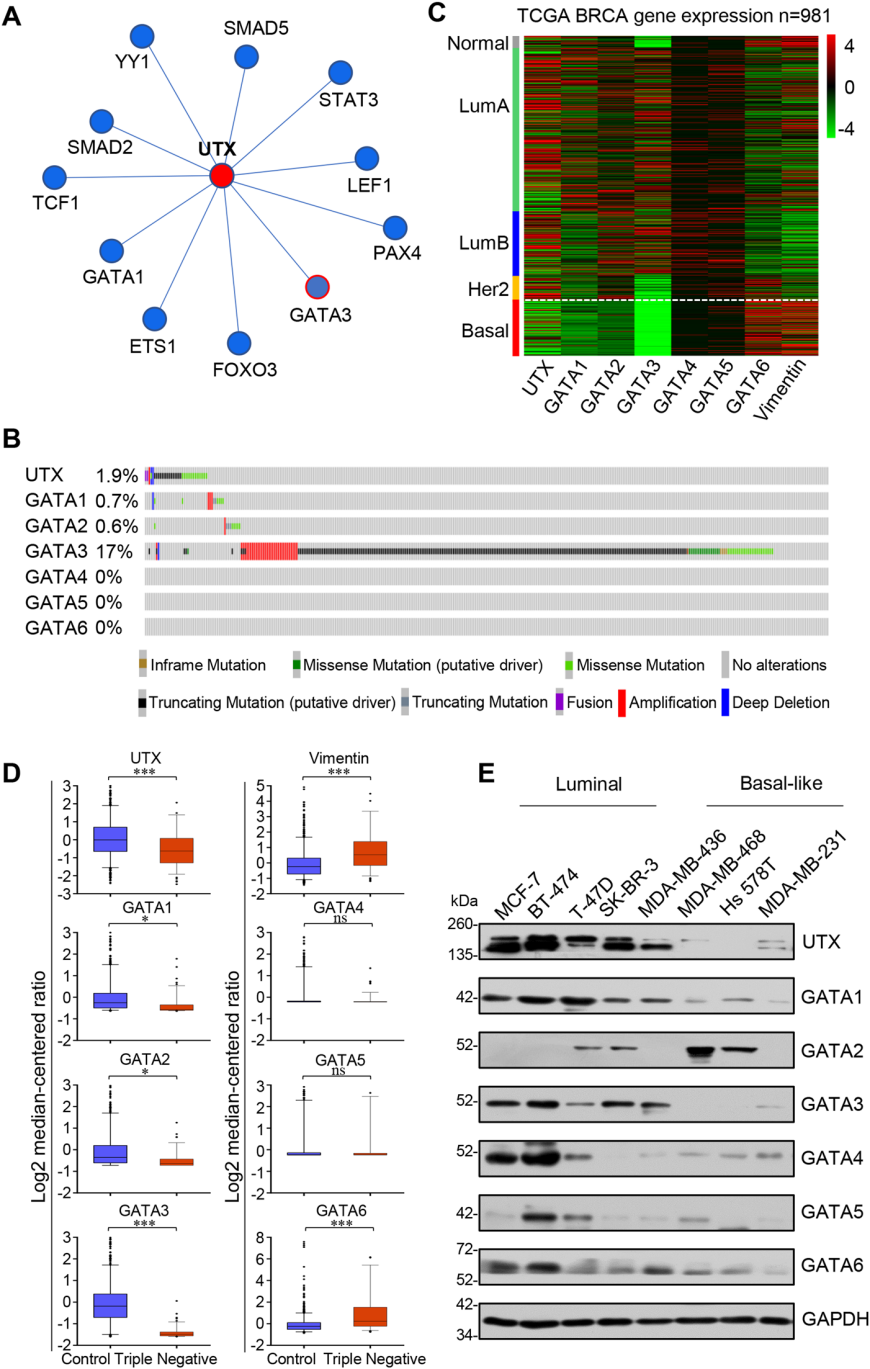
In silico analysis of breast cancer databases. **a** Analysis of GCBI database predicted that UTX is regulated by the transcription factor GATA3. **b** Analysis of 1918 patient samples from the TCGA database using cBioportal indicates that GATA3 expression was altered in 17% of the cases and that UTX is altered in 1.9% of the cases, with most of the UTX mutations occurring in cases lacking GATA3 mutation. **c** Analysis of TCGA database. Heatmap showing gene expression analysis of the GATA family, UTX, and Vimentin in distinct types of breast cancer (*n* = 981). **d** Analysis of GATA family, UTX, and Vimentin expression in breast cancer by using TCGA database. Triple-negative breast cancers expressed GATA3 and UTX at lower levels than the other subtype; Control: *n* = 987; Triple negative: *n* = 117 (*p < 0.05, **p < 0.01, ***p < 0.001; two-tailed unpaired *t* test). **e** GATA3 and UTX expression was elevated in luminal breast cancer cells, with GATA3 expression being positively correlated with UTX levels. Lysates from different human breast cancer cell lines were subject to western blotting to examine UTX and GATA-family protein levels

GCBI database analysis using Regulome Explorer (https://www.gcbi.com.cn/) predicted that UTX is regulated by the transcription factor GATA3, GATA1, and several other key transcription factors such as YY1, FOXO1, SMAD5, and SMAD2 (Fig. 1a), this finding suggests that GATA transcription factor expression could be linked to UTX levels in breast cancer. Analysis of 1918 patient samples from the TCGA database through cBioportal (http://www.cbioportal.org/) indicates that GATA3 expression was altered in 17% of the cases, these alterations are caused by gene amplification, mutation, deletion, and fusion, and among these, several missense and truncation mutations are considered to be potential driver mutations involved in breast cancer. UTX was altered in 1.9% of the breast cancer patients, with the mutations occurring mostly in cases where no GATA mutation was present (Fig. 1b). These data suggest that GATA3 and UTX complement each other in breast cancer cells to regulate tumorigenesis, with changes in either molecule potentially triggering the occurrence of breast cancer. Further RNA-sequencing (RNA-seq) analysis of a TCGA dataset composed of 981 patient samples suggests that UTX and GATA3 were expressed similarity at substantially lower levels in the samples of basal-subtype carcinomas than luminal-subtype carcinomas. To demonstrate the validity of our analysis, we also analyzed the pro-oncogene Vimentin, which has been reported to be elevated in various epithelial tumors and is one of the most prominent EMT biomarkers^20^, and its expression was upregulated in the absence of UTX expression (Fig. 1c). Moreover, comparison of the expression of UTX, Vimentin, and GATA transcription factor family in normal and triple-negative breast cancer samples using the TCGA database also show marked downregulation of GATA3 and UTX, whereas GATA6 was significantly upregulated (Fig. 1d). To further explore the relevance of the UTX and GATA transcription factors, the expression level of UTX and GATA1-6 were determined using western blotting analysis of luminal and basal-like breast-carcinoma cell lines. The results show that UTX levels were markedly higher in luminal cells than in basal-like cells, and GATA3 expression was highly positively correlated with that of UTX (Fig. 1e). These results suggest that GATA3 and UTX may be more closely related in breast cancer.

### GATA3 is physically associated with the UTX/MLL4 complex

**Fig. 2:**
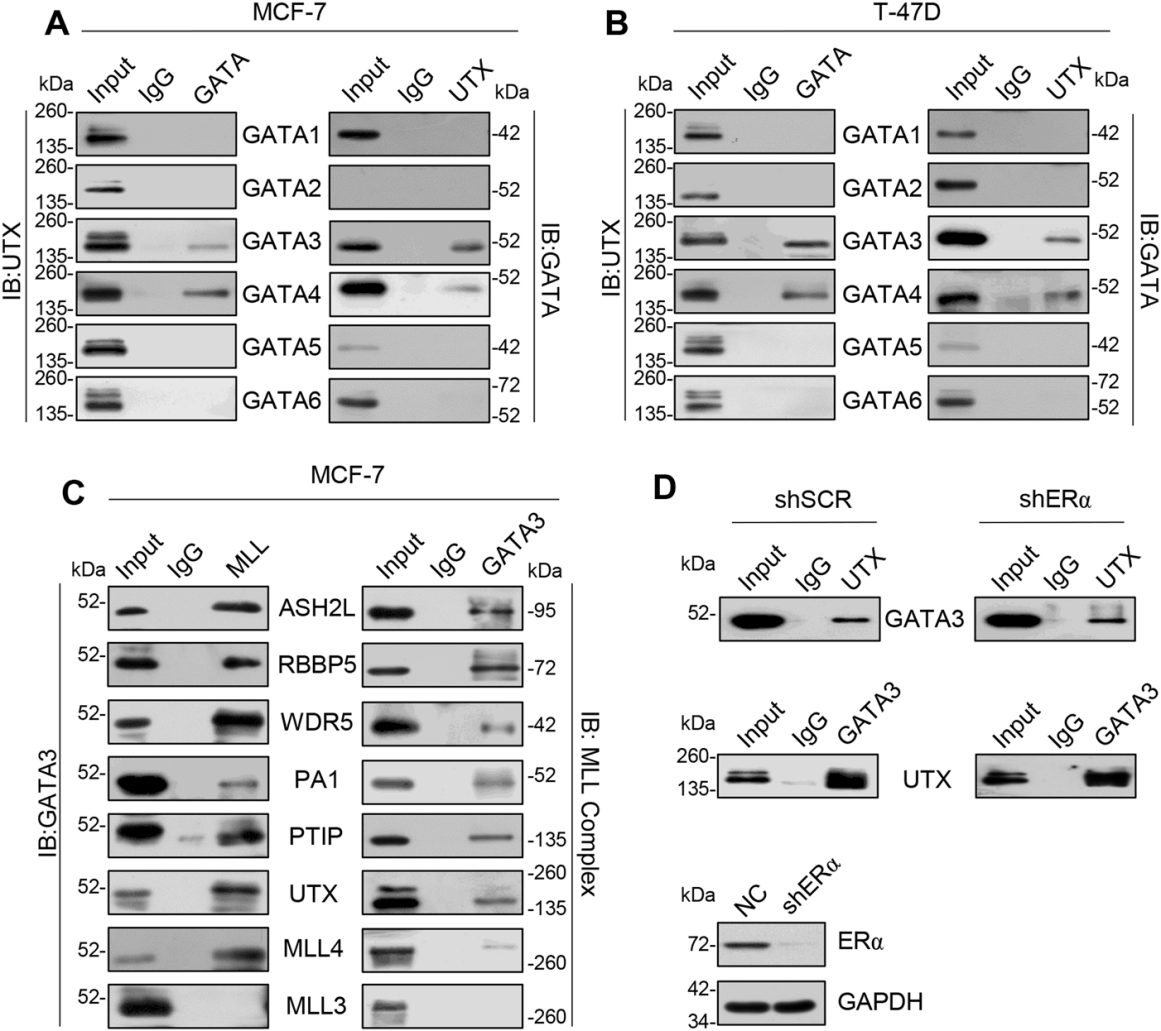
GATA3 is physically associated with UTX/MLL4 complex. **a**, **b** Association of UTX with GATA3 in MCF-7 and T-47D cells. Whole-cell lysates were prepared, and co-IP was performed using antibodies against GATA family or UTX, and then captured samples were immunoblotted with antibodies against the indicated proteins. IgG served as the negative control. **c** Association of GATA3 with MLL3/MLL4 complex in MCF-7 cells. Whole-cell lysates were immunoprecipitated with antibodies against GATA3, MLL3, or MLL4-complex proteins and immunocomplexes were immunoblotted with antibodies against the indicated proteins. **d** Interaction between GATA3 and UTX is independent of ERα. Whole-cell lysates were prepared from MCF-7 cells and co-IP was performed using antibodies against GATA3 or UTX, after which IB was performed with antibodies against the indicated proteins to examine the interaction in the presence and absence of ERα

To enhance our understanding of the mechanistic relationship between UTX and the GATA family, total proteins from MCF-7 cells were extracted, and coimmunoprecipitation (co-IP) assays were performed. Immunoprecipitates (IPs) with antibodies against GATA proteins were subjected to immunoblotting (IB) with antibodies against UTX, which show that GATA3 and GATA4 could physically interact with UTX. Reciprocally, IPs with antibodies against UTX followed by IBs with antibodies against GATA1-6 also confirmed these interactions (Fig. 2a). In addition to the association between UTX and GATA3, GATA4 was also detected in T-47D cells (Fig. 2b). The results of bioinformatics analyses revealed a close correlation between GATA3 and UTX, and GATA3 has emerged as a strong predictor of tumor differentiation and clinical outcome in breast cancer;^1,21^ therefore, we focused on the relationship between GATA3 and UTX. Because UTX is a subunit of the MLL3/MLL4 complex, the observed physical interaction between UTX and GATA3 led us to investigate potential crosstalk between MLL3/MLL4 complex and GATA3. We found that MLL4 rather than MLL3 could be readily co-immunoprecipitated with GATA3 (Fig. 2c). To further validate the interaction between GATA3 and the MLL4 complex in breast cancer cells, MCF-7 cell extracts were immunoprecipitated with antibodies against ASH2L, RBBP5, WDR5, PA1, PTIP, UTX, and MLL4. The IB of these samples revealed the co-IP of GATA3; moreover, reciprocal IPs with anti-UTX followed by IB with anti-MLL4-complex antibodies confirmed the association between these proteins (Fig. 2c). Because both MCF-7 and T-47D are ER^+^ breast cancer cell lines, and GATA3 and UTX are almost absent in ER^-^ breast cancer MDA-MB-231 cells, we suspected that the interaction between GATA3 and UTX does not depend on ERα. To test this, we prepared whole-cell lysates from MCF-7 cells and performed co-IP experiments in the presence and absence of ERα: IPs with anti-UTX followed by IB with anti-GATA3 antibodies detected the interaction of GATA3 with UTX in the cell lysates both in the presence and absence of ERα (Fig. 2d); this ERα-independent interaction was again confirmed in assays with IPs with antibody against GATA3 and IB with anti-UTX. Collectively, these results support the conclusion that the interaction between GATA3 and the UTX/MLL4 complex does not require ERα.

### Molecular interactions between GATA3 and UTX/MLL4 complex

**Fig. 3:**
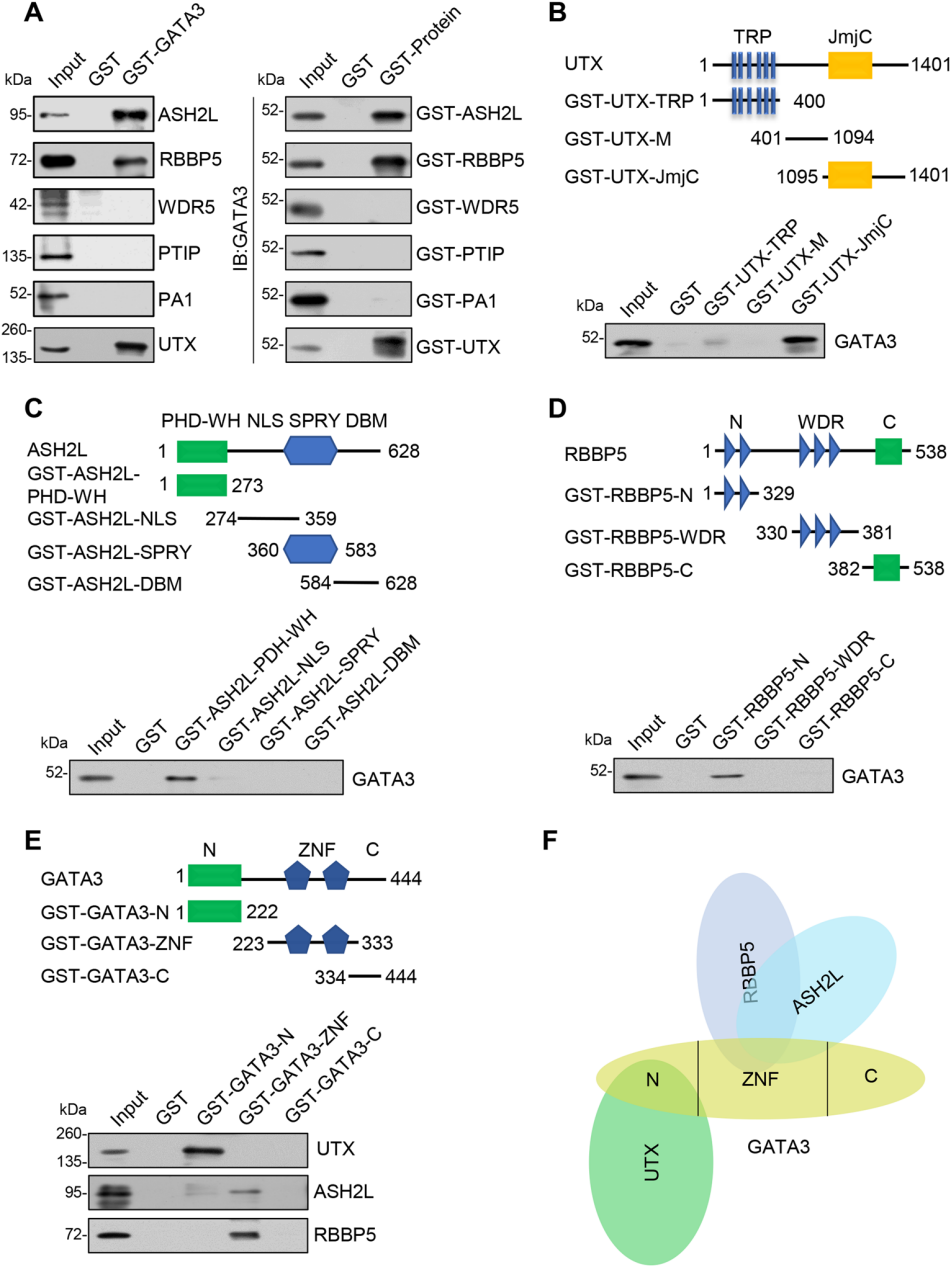
Molecular interaction between GATA3 and UTX/MLL4 complex. **a** GST pull-down experiments were performed using bacterially expressed GST-fusion proteins and proteins prepared using in vitro transcription/translation, as indicated. **b** GATA3 directly interacts with JmjC domain of UTX in vitro. GST pull-down assays were performed using a series of truncation constructs of UTX and in vitro transcribed/translated GATA3. **c** GATA3 directly interacts with PHD-WH domain of ASH2L in vitro. GST pull-down assays were conducted using a series of truncation constructs of ASH2L and in vitro transcribed/translated GATA3. **d** GATA3 directly interacts with N-terminal region of RBBP5 in vitro. GST pull-down assays were performed using a series of truncation constructs of RBBP5 and in vitro transcribed/translated GATA3. **e** Identification of GATA3 domains required for interaction with UTX, ASH2L, or RBBP5. GST pull-down assays were conducted using a series of truncation constructs of GATA3 and in vitro transcribed/translated proteins, as indicated. **f** Schematic depiction of molecular interactions between GATA3 and MLL4 complex

To gain insights into the molecular basis for the interaction between GATA3 and UTX/MLL4 complex, GST pull-downs were first performed using GST-fused GATA3 and in vitro transcribed/translated ASH2L, RBBP5, WDR5, PTIP, PA1, and UTX, which revealed that GATA3 can interact directly with UTX, ASH2L, and RBBP5; moreover, similar results were obtained in reciprocal GST pull-down assays (Fig. 3a). Furthermore, mapping of the interaction interface in UTX by using GST-fused UTX-domain constructs and in vitro transcribed/translated GATA3 revealed that the Jumonji C (JmjC) domain of UTX is responsible for interaction with GATA3 (Fig. 3b). Next, GATA3 interaction with ASH2L was dissected by using GST-fused PHD-WH, NLS, SPRY, and DBM domains of ASH2L, which revealed the binding of ASH2L-PHD-WH domain to GATA3 (Fig. 3c). Analogously, the N-terminal region of RBBP5 was found to be responsible for interaction with GATA3 (Fig. 3d). GATA3 contains two zinc-finger domains, and to determine which domain mediates the interactions of GATA3, we used a series of truncation constructs (GATA3-N, GATA3-ZnF, and GATA3-C) to generate GST-fusion proteins (Fig. 3e) for pull-down assays; our results show that the N-terminus of GATA3 (GATA3-N) is essential for UTX binding and that the GATA3 zinc-finger domain (GATA3-ZnF) is responsible for interaction with ASH2L and RBBP5 (Fig. 3e). Taken together, these results further supported the physical interaction between GATA3 and the MLL4 complex and revealed the molecular basis for the formation of the GATA3/UTX/MLL4 complex (Fig. 3f).

### Transcriptome analysis of GATA3/UTX-linked gene regulation in breast cancer cells

**Fig. 4:**
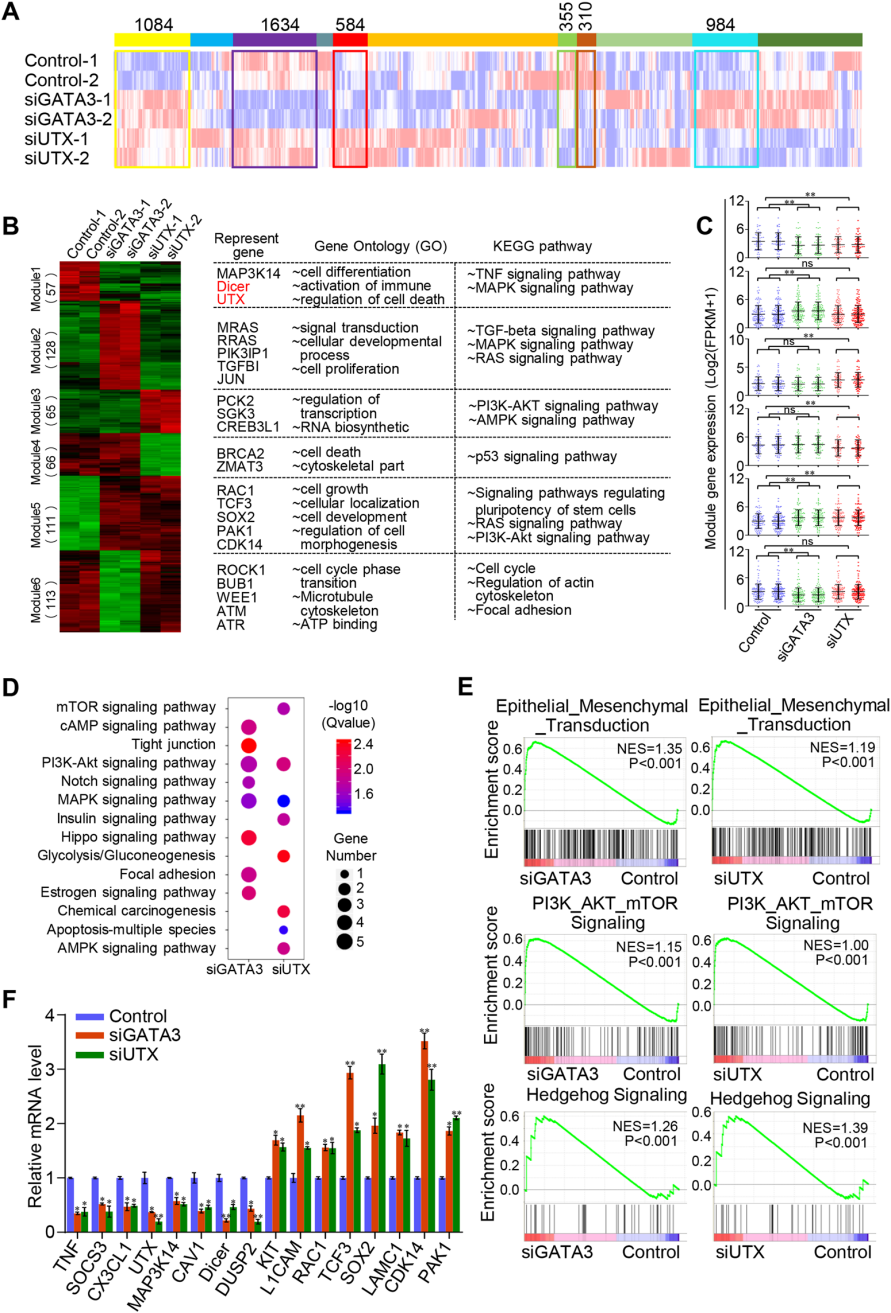
RNA-seq analysis of depletion of GATA3- or UTX-linked gene expression in MCF-7 cells. **a** Global transcriptome analysis of all detected genes (fragments per kilobase of transcript per million mapped reads [FPKM] > 0 in all samples). GATA3 or UTX was knocked down in MCF-7 cells by using siRNAs. Two independent samples were separately subject to RNA-seq analysis. Genes exhibiting specific expression patterns in the two experimental groups are highlighted in a colored box and the number of genes in each cluster is labeled. **b** Heatmap of gene expression from six representative modules. Representative genes, GO terms, and KEGG pathways of each module are also shown. **c** Box plot showing the FPKM of gene expression distribution of different modules. Targeted groups are compared with two control samples for each sample separately (*p < 0.05, **p < 0.01, ***p *<* 0.001; two-tailed unpaired *t* test). **d** Pathway analysis of GATA3/UTX-regulated target genes arranged into functional groups. **e** GSEA results indicating that GATA3- and UTX-knockdown groups showed significant enrichment of common differentially expressed genes in several critical cellular processes. **f** Verification of RNA-seq results through qPCR analysis of the indicated genes in MCF-7 cells. Results are represented as fold-change over control, with GAPDH used as the internal reference. Data are shown as means ± SD from three independent experiments (*p < 0.05, **p < 0.01, ***p < 0.001; two-tailed unpaired *t* test)

To delineate the molecular pathways that depend on GATA3 and UTX complex, we conducted RNA-seq analysis on in vitro cultured cells. In these experiments, siRNA was used to knockdown the expression of GATA3 or UTX in MCF-7 cells. After RNA extraction, purification, reverse transcription (RT), and amplification, the DNA product was cyclized and sequenced. Two independent samples and controls were used in these experiments. The detailed results of the RNA-seq experiments are summarized in Supplementary File 1. Whole-transcriptome clustering analysis revealed that 1084 genes (6.31%) were co-upregulated whereas 310 genes (1.81%) were co-downregulated in the siGATA3 and siUTX groups (Fig. 4a). To further dissect the regulation dependent on the GATA3/UTX complex, unsupervised hierarchical clustering analysis was used to identify distinct co-expression modules in the tested groups. We focused here on 6 modules that distinguish the three experimental groups based on specific expression patterns (Fig. 4b): module 1 (*n* = 57) and module 5 (*n* = 111) were two groups of co-altered genes in both GATA3-knockdown groups and UTX-knockdown groups. To investigate the signaling cascades downstream of the GATA3/UTX complex, we analyzed the gene ontology (GO) and Kyoto Encyclopedia of Genes and Genomes (KEGG) pathways for each module and found that the TNF and MAPK signaling pathways were enriched in the dysregulated genes identified in GATA3- and UTX-knockdown groups (Fig. 4b). Subsequently, the gene expression level was normalized based on quartile normalization and the generated box plot shows the expression level of each module, which indicated that the expression of specific module genes in a particular distribution group agreed with the heatmap results (Fig. 4c). The differentially expressed genes targeted by GATA3 or UTX were then classified into various cellular signaling pathways using KEGG pathway analysis (Fig. 4d). The results show that both siGATA3 and siUTX group were involved in the PI3K-Akt, MAPK, and AMPK signaling pathways. Moreover, the differentially expressed genes were compared pairwise by using the gene set enrichment analysis (GSEA) approach; the siGATA3 or siUTX groups, but not Control groups, showed significant enrichment of differentially expressed genes in several key cellular processes, such as EMT, PI3K/Akt/mTOR-signaling, and Hedgehog-signaling pathways (Fig. 4e), which are highly associated with cell proliferation and metastasis. In accord with the RNA-seq results, knockdown of GATA3 or UTX in MCF-7 cells led to altered expression of several crucial genes at the transcriptional level (Fig. 4f); these genes included the gene encoding RAC1, a vital transcription factor closely related to EMT and cell invasion^22,23^, and Dicer, a tumor suppressor that plays a critical role in miRNA-pathway-mediated posttranscriptional gene silencing^24,25^.

### Genome-wide identification of transcriptional targets of GATA3/UTX complex

**Fig. 5:**
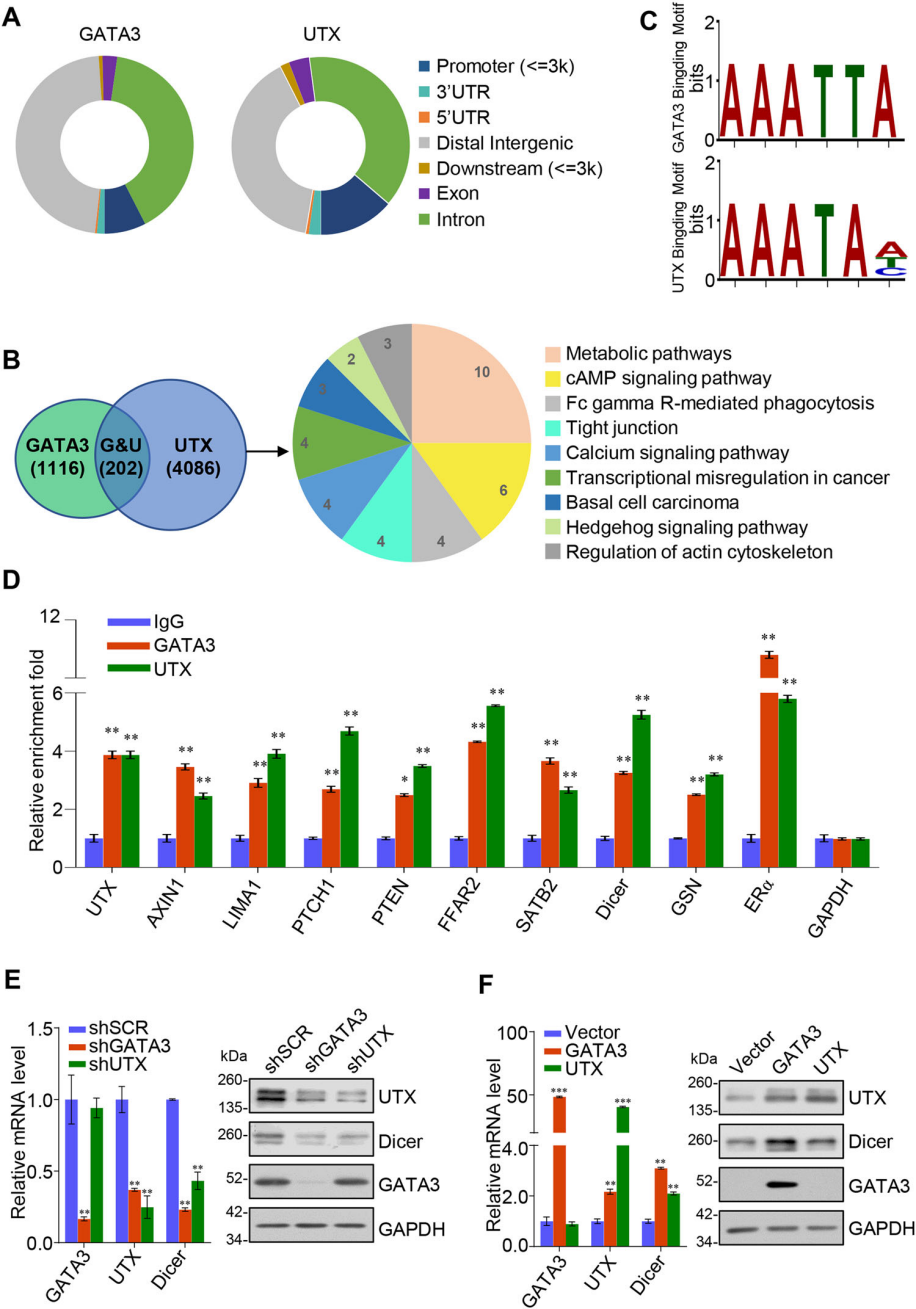
Genome-wide transcriptional-target analysis for GATA3/UTX/MLL4 complex. **a** Genomic distribution of GATA3 and UTX targets, based on ChIP-seq analysis. **b** Left: Venn diagram showing overlapping promoters bound by GATA3 and UTX in MCF-7 cells. Right: pathway analysis of 202 overlapping target genes of GATA3 and UTX arranged into functional groups. **c** MEME-ChIP analysis of GATA3- and UTX-bound motifs. **d** Verification of ChIP-seq results through qChIP analysis of indicated genes in MCF-7 cells. Results are expressed as fold-change relative to control, and GAPDH was used as a negative control. Data are shown as means ± SD from three independent experiments. **e** qPCR and western blotting analysis of the expression of UTX and Dicer in GATA3- or UTX-depleted MCF-7 cells. **f** qPCR and western blotting analysis of the expression of UTX and Dicer in GATA3- or UTX-overexpressing MDA-MB-231 cells. **d**–**f** Two-tailed unpaired *t* test (*p < 0.05, **p < 0.01, ***p < 0.001)

We further investigated the functional association between GATA3 and UTX by analyzing the genome-wide distribution of their targets. Here, the ChIP experiments were conducted using MCF-7 cells and antibodies against GATA3 or UTX, and then after ChIP, GATA3- and UTX-associated DNAs were amplified using non-biased conditions, labeled, and sequenced on the HiSeq 2000 platform. The detailed results of the ChIP-seq experiments are summarized in Supplementary File 2. Using a *p*-value cutoff of 10^–3^, we identified 15647 GATA3-specific binding peaks and 34583 UTX-specific binding sites (Fig. 5a). The data from the GATA3 and UTX groups were then analyzed for overlapping DNA sequences/gene promoters, and these promoters were considered to be the targets of the GATA3/UTX complex. Our results identified 202 promoters targeted by both GATA3 and UTX. The genes corresponding to these promoters were classified into various cellular signaling pathways by using KEGG pathway software. These signaling pathways include metabolic-, cAMP-, and Hedgehog-signaling pathways, which are all critically involved in cell growth, migration, and invasion (Fig. 5b). Notably, we found that GATA3 and UTX featured similar binding motifs (Fig. 5c). Next, qChIP analysis was performed using MCF-7 cells and specific antibodies against GATA3 and UTX for selected genes, including the genes encoding *UTX, AXIN1, LIMA1, PTCH1, PTEN, FFAR2, SATB2, Dicer, GSN*, and *ERa*; the results show a strong enrichment of GATA3 and UTX on the promoters of these genes, validating the ChIP-seq results (Fig. 5d). To further explore the transcriptional regulation of GATA3/UTX on these target promoters, qPCR and western blot experiments were performed on two representative target genes, *UTX* and *Dicer*. Both genes typically carry deletions or mutations during tumor progression, and their loss of function is recognized as a driver of tumorigenesis^17,26^. The qPCR and western blotting results for UTX and Dicer in MCF-7 cells show that their expression was notably diminished after knockdown of GATA3 or UTX, but UTX depletion did not cause a decrease in GATA3 expression (Fig. 5e); accordingly, overexpression of GATA3 or UTX led to the induction of UTX and Dicer at both mRNA and protein levels (Fig. 5f). These data were also consistent with the analysis of RNA-seq (Fig. 4b). Collectively, these results suggest that GATA3 recruits UTX and transcriptionally activates the downstream target genes UTX and Dicer, supporting the physical interaction between GATA3 and UTX.

### GATA3 and UTX inhibit EMT, invasion, and metastasis of breast cancer cells

**Fig. 6:**
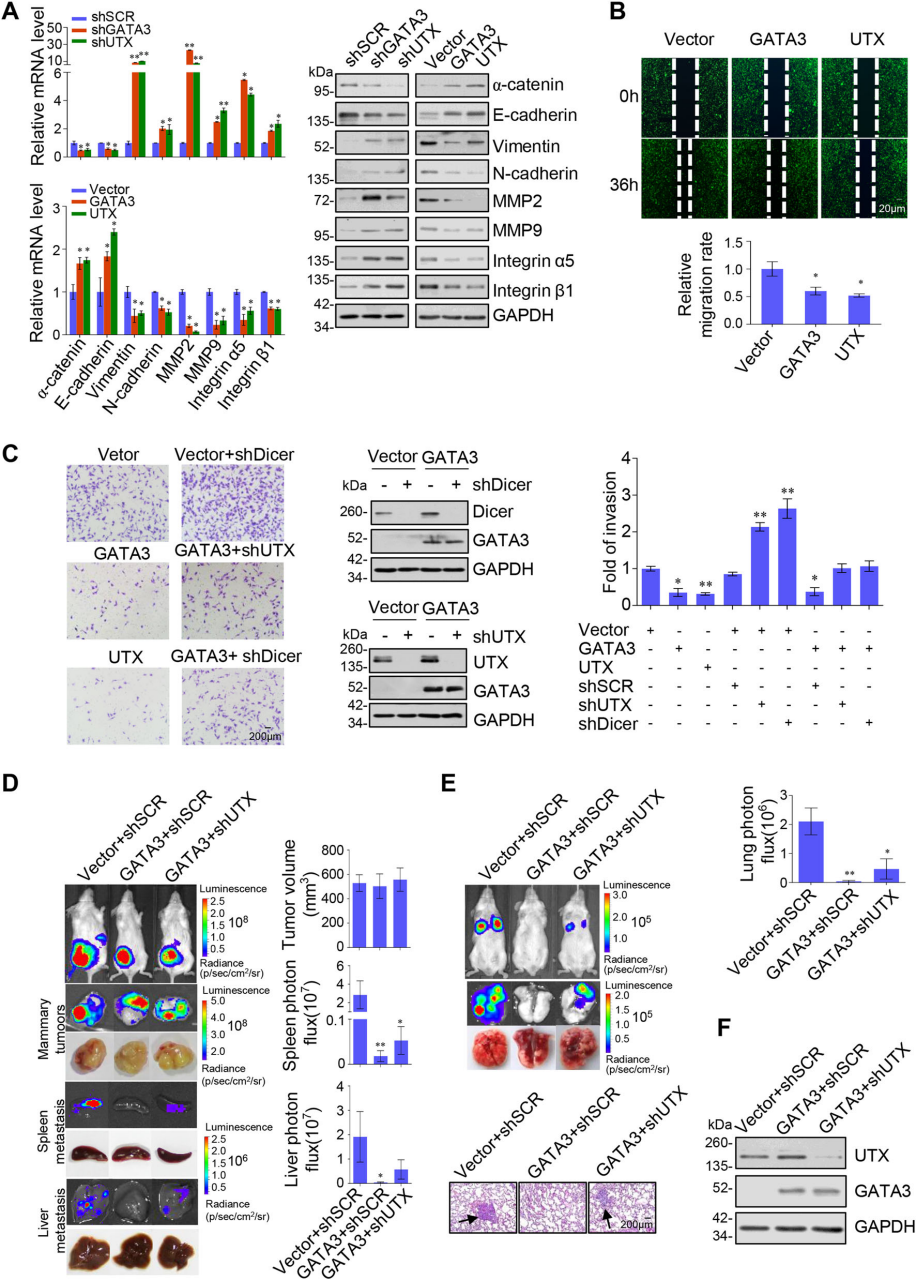
GATA3/UTX complex inhibits metastasis potential of breast cancer in vitro and in vivo. **a** Expression of epithelial, mesenchymal, invasion, and metastatic markers was measured using RT-PCR (left) and western blotting (right) in GATA3/UTX-depleted MCF-7 cells and GATA3/UTX-overexpressing MDA-MB-231 cells. **b** GATA3/UTX-overexpressing MDA-MB-231 cells were subjected to linear wounds in the cell monolayer, and after 36 h, the cell migration into the wound was examined under an optical microscope and then relative mobility was estimated‘. **c** MDA-MB-231 cells were transfected with the indicated specific shRNA and/or expression constructs and used in cell-invasion assays. Invaded cells were stained and counted (right). The image represents one field of view under the microscope in each group (left). Western blotting was used to verify the efficiency of protein knockdown or overexpression (middle). **d** MDA-MB-231-Luc-D3H2LN cells infected with lentiviruses carrying the indicated expression constructs and/or shRNAs were inoculated orthotopically into the abdominal mammary fat pad of 6-week-old female SCID mice (*n* = 6). Primary tumors and metastases were quantified through bioluminescence imaging at 8 weeks after initial implantation. Representative in vivo bioluminescence images are shown, and tumor specimens were examined using in vitro bioluminescence measurement. **e** The MDA-MB-231-Luc-D3H2LN cells described above were injected intravenously through the tail vein of 6-week-old female SCID mice (*n* = 6). Lung metastasis was quantified using bioluminescence imaging after 8 weeks. Representative in vivo bioluminescent images are shown. Lung tumor specimens were examined using in vitro bioluminescent measurement and representative lung metastasis specimens were sectioned and stained with H&E (left). **a**–**e** Error bars indicate mean ± SD (*p < 0.05, **p < 0.01, ***p < 0.001; two-tailed paired *t* test). **f** The protein expression in MDA-MB-231-Luc-D3H2LN cells was confirmed by western blotting

Next, we investigated the role played by GATA3 and UTX in breast cancer invasion and metastasis: we performed GATA3/UTX loss-of-function and gain-of-function experiments and examined the expression levels of epithelial and mesenchymal markers (Fig. 6a). Depletion of either GATA3 or UTX in MCF-7 cells resulted in reduced expression of epithelial markers, including α-catenin and E-cadherin, at both mRNA and protein levels, increased expression of mesenchymal markers, including N-cadherin, Vimentin, and increased expression of invasion and metastatic markers such as MMP2, MMP9 Integrin α5, and Integrin β1. Conversely, overexpression of GATA3 or UTX led to elevation of epithelial markers and reduction of mesenchymal, invasion, and metastatic markers. We next investigated the roles of GATA3 and UTX in the migration of breast cancer cells in vitro by using the wound-healing assay; overexpression of GATA3 or UTX resulted in a notable delay in wound closure as compared with the control (Fig. 6b). Moreover, the results from transwell assays performed using MDA-MB-231 cells show that GATA3 or UTX overexpression caused a substantial reduction of cell invasion, whereas depletion of UTX or Dicer individually resulted in an increase of the invasive potential of MDA-MB-231 cells (Fig. 6c). Furthermore, inhibition of the invasive potential of MDA-MB-231 cells associated with GATA3 overexpression could be diminished by reducing the expression of UTX, and, similarly, the reduction of Dicer expression also caused the same results (Fig. 6c). These data indicate that the GATA3/UTX complex is essential for the epithelial homoeostasis of breast cancer cells owing to its activation of the suppressor genes *Dicer* and *UTX* to form a GATA3-UTX-Dicer axis.

To investigate the role of the GATA3 and UTX in breast cancer metastasis in vivo, MDA-MB-231 cells that stably express firefly luciferase (MDA-MB-231-Luc-D3H2LN, Xenogen) were co-infected with lentiviruses carrying empty vector + shSCR, GATA3 + shSCR, or GATA3 + shUTX. The cells were then orthotopically implanted into the abdominal mammary fat pad or intravenously injected into the lateral tail vein of 6-week-old female immunocompromised with severe combined immunodeficiency (SCID) mice to measure spontaneous metastasis or seeding lung metastasis, respectively. Primary tumor growth and tumor metastasis were monitored weekly through bioluminescence imaging performed using an IVIS imaging system (Xenogen Corporation). Tumor metastasis was measured using quantitative bioluminescence imaging after 8 weeks for the orthotopically implanted and intravenous injection groups. A metastatic event was defined as any detectable luciferase signal that was above the background signal and distant from the original tumor site. For the orthotopically implanted groups, the control MDA-MB-231-Luc-D3H2LN cells, metastases were generated after 8 weeks. GATA3 overexpression did not affect the growth of primary tumors but led to a marked reduction in liver and spleen metastases; notably, although UTX depletion also produced no effect on the growth of primary tumors, the depletion partially abrogated the metastasis inhibition associated with GATA3 overexpression (Fig. 6d). Similarly, in the intravenous injection groups, GATA3 overexpression led to a dramatic decrease in lung metastasis of the MDA-MB-231-Luc-D3H2LN tumors, and the suppressive effect of GATA3 overexpression on lung metastasis was, at least partially, abolished when UTX was knocked down (Fig. 6e). Metastases to the lungs were measured using bioluminescence imaging and histological staining (Fig. 6e), and the efficiency of lentivirus-mediated overexpression or knockdown in MDA-MB-231-Luc-D3H2LN cells was verified using western blotting (Fig. 6f). In conclusion, these experiments indicate that GATA3 recruits UTX to suppress breast cancer metastasis, supporting a role for the GATA3/UTX complex in controlling the metastasis of breast cancer in vivo.

### Clinicopathological significance of GATA3-UTX-Dicer in breast cancer

**Fig. 7:**
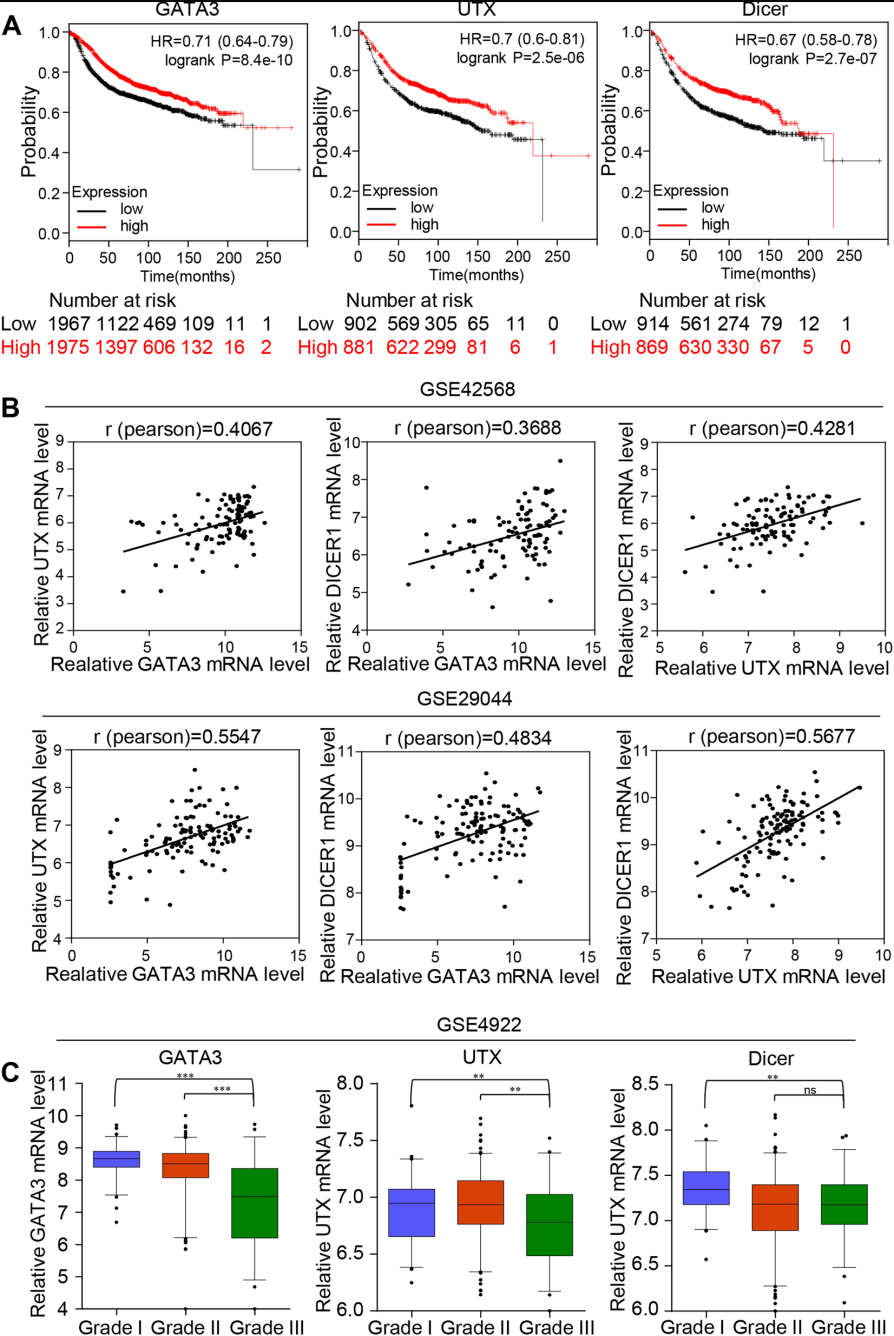
Clinicopathological significance of GATA3-UTX-Dicer axis in breast cancer. **a** Kaplan–Meier survival analysis of the relationship between survival time and GATA3, UTX, and Dicer expression in breast cancer, performed using an online tool (http://kmplot.com/analysis/). **b** Analysis of public datasets (GSE42568 and GSE29044) for expression of GATA3, UTX, and Dicer in breast cancer. Relative levels of UTX or Dicer are plotted against GATA3 levels, and the relative levels of GATA3 are plotted against UTX levels. **c** Analysis of public datasets (GSE4922) for expression of GATA3, UTX, and Dicer in breast carcinomas with histological grades I, II, and III (*p < 0.05, **p < 0.01, ***p < 0.001; two-tailed unpaired *t* test)

We analyzed the correlation between the expression of GATA3, UTX, and Dicer and the clinical outcomes of patients with breast cancer: Kaplan–Meier survival analysis performed in relation to GATA3, UTX, and Dicer by using an online tool (http://kmplot.com/analysis/) revealed that elevated expression of any of the 3 molecules was associated with enhanced overall survival of breast cancer patients (Fig. 7a). To obtain further support for the role of the GATA3-UTX-Dicer axis in the progression of breast cancer and to study the clinicopathological significance of this axis, we analyzed two published clinical datasets (GSE42568 and GSE29044) from the GEO database (http://www.ncbi.nlm.nih.gov/geo/). Our results show that GATA3 and UTX expression levels positively correlated not only with each other, but also with the expression of Dicer (Fig. 7b). To further investigate the role of GATA3, UTX, and Dicer in breast cancer progression, GSE4922 from the GEO database was used to analysis the relationship between the GATA3-UTX-Dicer axis and histological grades. As shown in Fig. 7c, the expression of GATA3, UTX, and Dicer was found to be negatively correlated with histological grades. These results bolster the view presented here regarding the function of the GATA3-UTX-Dicer axis. In summary, our analysis results show that GATA3, UTX, and Dicer are downregulated in breast cancer, and UTX and Dicer might represent potential breast cancer biomarkers.

## Discussion

GATA3 downregulation has been observed in ER-negative breast cancers and is associated with favorable disease-free and overall survival rates in the case of patients with breast cancer^27^. GATA3 has been reported to function in both transcription repression^21^ and activation^28^. In the mammary gland, GATA3 and ERα regulate each other and bind to FOXA1, which can nucleate recombination complexes in the heterochromosomal region of ERα target genes; this results in the opening and epigenetic marking of active transcriptional sites^29,30^. By contrast, in breast cancer cells, GATA3, G9A, and NuRD (MTA3) act in a coordinated manner to form a transcription-repression complex that regulates mammary epithelial plasticity by controlling EMT-related gene expression^21^. These findings indicate that GATA3 performs disparate functions in the mammary gland and in breast cancer depending on differential interaction with specific cofactors in the given genetic/epigenetic context.

UTX was first identified as a component of the MLL4/COMPASS complex^31^ and was reported to be highly mutated in distinct human cancers^19,32,33^. UTX somatic mutation has been found to be potentially related to its loss of function;^19^ and in human breast cancer, UTX is a central factor mediating EMT^34,35^. These findings all provide insights into the mechanistic function of UTX as a tumor suppressor. We show that the UTX was recruited by GATA3, and the results of our RNA-seq and ChIP-seq experiments show that the GATA3/UTX complex can co-regulate diverse target genes; moreover, GSEA results revealed an enrichment of EMT, PI3K/Akt/mTOR-signaling, and Hedgehog-signaling gene signatures following downregulation of GATA3 or UTX. These results suggest that UTX could act as a tumor suppressor to influence the invasion and metastasis of breast cancer, which agrees with a previous report that UTX suppresses EMT-induced cancer-stem-cell properties in breast cancer^36^.

The findings that GATA3 physically interacts with the UTX/MLL4 complex suggest that the tumor suppressive role of this complex might be ensured through the maintenance of a proper transcriptional-network activation by eliminating PRC2-induced putative oncogenic silencing. We suspect that GATA3 recruits UTX/MLL4, allowing them to function in a coordinated manner, either simultaneously or sequentially methylating H3K4 and demethylating H3K27, to broadly maintain increased level of H3K4me3^37^ and decreased level of H3K27me2/3 at the target promoters, thereby transcriptionally activating tumor suppressor genes such as UTX or Dicer and inhibiting the progression of breast cancer. However, whether the function of the GATA3/UTX complex depends on enzymatic activity remains to be further studied.

Our GATA3 and UTX ChIP-seq results combined with the RNA-seq results revealed that the GATA3/UTX complex directly activates a collection of genes that includes *Dicer* gene and *UTX* gene itself. Dicer is a member of the RNase III family and is a key factor in the biogenesis of small-regulatory RNAs (including miRNA and siRNA). Dicer is a haploinsufficient tumor suppressor that plays a pivotal role in inhibiting tumor metastasis, and under conditions of <50% repression, Dicer drives tumorigenesis^24–26^. FoxO3 has been reported to transcriptionally activate Dicer, which in turn regulates miRNA maturation and breast cancer metastasis^25^. Moreover, hypoxia is associated with reduced expression of Dicer in breast cancer patients^38^. Further investigation is required to determine whether the results of these studies are related to changes in miRNA synthesis induced by the GATA3-UTX-Dicer axis.

Interestingly, we found that UTX itself is a target of the GATA3/UTX complex. Thus, a feedback loop between UTX and the GATA3/UTX complex appears to exist in mammary tissue, wherein GATA3 transactivates UTX, which is assembled into the GATA3/UTX complex, and this complex, in turn, transactivates UTX. In summary, this study revealed that GATA3 recruits the UTX to form a complex in breast cancer cells, which provides new insights into how the UTX selects its interacting transcription factors in breast cancer cells. Our results show that the GATA3-UTX-Dicer axis is involved in breast cancer metastasis, and further that elevated expression of GATA3, UTX, and Dicer is correlated with favorable prognosis in breast cancer. Our data provide a molecular basis for understanding the pathophysiological function of GATA3 and support the view that the GATA3-UTX-Dicer axis can serve as a potential therapeutic target in breast cancer.

## Materials and methods

### Cell culture and transfection

The cell lines used here were all originally purchased from American Type Culture Collection (Manassas, VA, USA) and were authenticated in 2018 by means of short-tandem-repeat analysis. MCF-7 cells were cultured in Dulbecco’s modified Eagle’s medium (DMEM) supplemented with 10% fetal bovine serum and 1% antibiotics. MDA-MB-231 cells were cultured in L-15 medium supplemented with 10% fetal bovine serum and 1% antibiotics. T-47D cells were maintained in RPMI1640 medium. Cells were maintained in a humidified incubator equilibrated with 5% CO_2_ at 37 °C. siRNAs were transfected at a working concentration of 100 nM using Lipofectamine® RNAiMAX Reagent (Invitrogen, Carlsbad, CA, USA) according to the manufacturer’s instructions. Stable cell lines expressing GATA3, shGATA3, or shUTX were generated by transfecting pSG5-FLAG-GATA3, pGPU6-GFP-shGATA3, or pGPU6-GFP-shUTX into MCF-7 or MDA-MB-231 cells using the transfection reagent polybrene. The siRNA and shRNA sequences are listed in Supplementary File 3.

### Antibodies and reagents

Anti-Vimentin antibody (V6630) was purchased from Sigma-Aldrich, anti-N-cadherin (610920), anti-E-cadherin (610181), anti-MLL3 (53641), anti-α-catenin (610193), and anti-GATA3 (5852) from Cell Signaling Technology, anti-GAPDH (AC002) from ABclonal Technology, anti-UTX (A302-374A), anti-PA1 (A301-978A), anti-WDR5 (A302-429A), anti-PTIP (A300-370A), anti-ASH2L (A300-489A), and anti-RBBP5 (A300-109A) from Bethyl, anti-Dicer (ab14601) from Abcam, anti-MLL4 (C15310100) from Diagenode, anti-GATA1 (60011-1-Ig) and anti-GATA4 (19530-1-AP) from Proteintech, anti-GATA2 (sc-267) and anti-GATA5 (sc-373683) from Santa Cruz, and anti-GATA6 (AF1700) from R&D Systems. Dynabeads Protein G was obtained from Invitrogen (Thermo Fisher Scientific) and Glutathione-Sepharose 4B beads from GE Healthcare Bio-Sciences. shRNAs were synthesized by GenePharma Co. Ltd. (Shanghai, China). Glutathione S-transferase (GST)-MLL4-complex plasmids were created by inserting full-length MLL4-complex components into the pGEX-4T-3 expression vector as described previously^21^.

### Immunoprecipitation and western blotting

For immunoprecipitation (IP) assays, cells were washed twice with cold phosphate-buffered saline (PBS) and extracts were prepared by incubating cells in lysis buffer (50 mM Tris–HCl, pH 7.4, 150 mM NaCl, 1 mM EDTA, 0.5% NP-40, 0.25% sodium deoxycholate, and protease-inhibitor cocktail) for 30 min at 4 °C and then centrifuging at 12000 × *g* for 15 min. Next, 500-μg protein samples were incubated with appropriate primary antibodies or normal rabbit/mouse IgG at 4 °C for 12 h with constant rotation, and then mixed with glutathione-Sepharose beads for 2 h at 4 °C. After washing the beads 4 times with the cell-lysis buffer, the captured immune complexes were subject to SDS-PAGE followed by immunoblotting (IB) with secondary antibodies. Stained bands were detected using enhanced chemiluminescence (ECL System, Thermo Scientific) according to the manufacturer’s instructions.

### GST pull-down experiments

GST-fusion constructs were produced in *Escherichia coli* BL21 cells, and crude bacterial lysates were obtained by sonicating the cells in cold PBS supplemented with a protease inhibitor. In vitro transcription and translation experiments were performed using rabbit reticulocyte lysates (TNT Systems; Promega) according to the manufacturer’s instructions. In GST pull-down assays, 5–8 μL of the in vitro transcription/translation product was mixed with ~10 μg of the appropriate GST-fusion protein and incubated in binding buffer (0.8% BSA in PBS containing the protease inhibitor), after which 30 μL of glutathione-agarose beads were added to the binding-reaction solution and mixed by rotation at 4 °C for 2 h, washed five times with binding buffer, and then resuspended in 30 μL of 2 × SDS-PAGE loading buffer. Proteins were detected by means of western blotting performed using specific antibodies.

### RNA-sequencing analysis

Total RNA (1 mg) was extracted and purified using oligo(dT)-attached magnetic beads, and RNA quality was assessed on an Agilent 2100 Bioanalyzer. Next, mRNA molecules were fragmented into small pieces by using a fragmentation reagent, after which random-hexamer-primed reverse-transcription (RT) was performed to generate first-strand cDNA and then double-stranded cDNA. The synthesized cDNA was subject to end-repair and then 3ʹ-adenylated, and adapters were ligated to the ends of the cDNA fragments. Adapter-ligated libraries were generated by performing PCR with Illumina PE primers. The resulting cDNA libraries were applied onto an Illumina flow-cell for cluster generation (TruSeq cluster generation kit V.5) and sequenced. Genes with FPKM > 0 in all samples were retained for further analyses. Differentially expressed genes between each cell group with *Q* value <0.05 and fold-change >1.4 were identified.

### RT-PCR

Total cellular RNA was extracted from cells by using TRIzol, according to the manufacturer’s instructions (Invitrogen). Potential DNA contamination was mitigated by treating samples with RNase-free DNase (Promega). cDNA was prepared using MMLV Reverse Transcriptase (Roche). For amplification, the cDNA was mixed with 1 μL of forward and reverse primers (5 μM each), 5.5 μL of RNase-free water, and 7.5 μL of 2 × PCR SYBR Green Mix buffer in a 15-μL reaction. The PCR protocol comprised 40 cycles of 95 °C for 15 s and 60 °C for 1 min. Relative quantitation was performed by using an ABI PRISM 7500 sequence-detection system (Applied Biosystems, Foster City, CA, USA) and measuring real-time SYBR green fluorescence, and results were obtained by using the comparative Ct method (2-ΔΔCt) with glyceraldehyde 3-phosphate dehydrogenase (GAPDH) as an internal control. The primers used are listed in Supplementary File 3.

### Chromatin IP (ChIP), quantitative ChIP (qChIP), and ChIP-sequencing (ChIP-seq) assays

ChIP and qChIP assays were performed using MCF-7 cells as described previously^39^. Briefly, 1 × 10^7^ cells were crosslinked with 1% formaldehyde, sonicated, precleared, and incubated with 2–3 μg of primary antibody against normal rabbit IgG (control), GATA3, or UTX. The complex was washed with low-salt and high-salt buffers and then DNA was extracted for qChIP and ChIP-seq assays. The primers used for qChIP are listed in Supplementary File 3. For ChIP-seq, quantified 10 ng of DNA was resolved using an Agilent Technologies 2100 Bioanalyzer and 50–250-bp fractions were extracted, and the fractions were then subject to end-repair and 3ʹ-adenylation. Adapter-ligated libraries were amplified, purified, and selected using an Agencourt AMPure XP-Medium kit, and the final library was composed of single-stranded circular DNAs. In-depth whole-genome DNA sequencing was performed by CapitalBio Corporation (Beijing, China). Sequencing data acquired from the Illumina analysis pipeline were compared with the unmasked human reference genome hg19 (UCSC GRCh37) by using ELAND (Illumina, San Diego, CA, USA). The peaks were called using Model-based Analysis of ChIP-Seq (MACS) after filtering through the input. ChIPseeker was used to analyze genomic distribution of GATA3 or UTX binding sites.

### Wound-healing assay

After overexpression of GATA3 or UTX, cells were seeded into 6-well dishes and grown for 24 h to 80–90% confluence. A linear wound was created by scraping the subconfluent cell monolayer by using a pipette tip (200 μL, Axygen). After washing with PBS to remove cell debris, cell migration into the wound was monitored every 4 h, and following 36-h incubation at 37 °C, the migration of cells into the wound was examined under a light microscope to quantify the relative mobility of cells. The assays were performed at least thrice.

### Cell-invasion assay

Transwell-chamber filters (Becton Dickinson) were coated with Matrigel. Cells were infected with lentivirus, resuspended in serum-free medium, and pipetted into the upper chamber of the Transwell apparatus (at 3 × 10^4^ cells/0.5 mL of serum-free medium). The chamber was then transferred to a well containing 500 μL of medium supplemented with 10% fetal bovine serum, and the cells were incubated for 18–24 h at 37 °C. Subsequently, the cells in the upper well were removed by wiping the top of the membrane with a cotton swab, and the membrane was then stained with crystal violet and the cells on the lower surface of the membrane were counted. In the case of each membrane, the cells in three high-power fields were counted.

### In vivo metastasis

MDA-MB-231 cells that had been engineered to stably express firefly luciferase (Xenogen) were infected with lentiviruses carrying vector + shSCR, GATA3 + shSCR, or GATA3 + shUTX. A total 5 × 10^6^ cells of each type were inoculated into the left abdominal mammary fat pad or injected into the lateral tail vein (1 × 10^6^ cells) of 6-week-old female SCID mice. For bioluminescence imaging, mice were injected abdominally with 200 mg/g d-luciferin in PBS. At 10 min after injection, the mice were anesthetized and a charge-coupled device camera (IVIS; Xenogen) was used to image the bioluminescence. Bioluminescence images were obtained using these settings: field of view, 15 cm; binning (resolution) factor, 8; open filter; 1/f stop; imaging time, 30 s to 2 min. Bioluminescence from relative optical intensity was manually defined. The photon flux was normalized to the background, which was defined by the relative light intensity plotted from fluorescein-free mice.

### Statistical analysis

Results are reported as mean ± SD unless otherwise noted. Comparisons were performed using two-tailed unpaired *t* test. SPSS V.17.0 was used for statistical analysis.

## Supplementary information

Supplementary File 1

Supplementary File 2

Supplementary File 3
