# Supplementary material for: GATA3 recruits UTX for gene transcriptional activation to suppress metastasis of breast cancer
Source: Cell Death Dis. 2019 Nov 4;10(11):832. doi: 10.1038/s41419-019-2062-7 (PMC6828764; doi:10.1038/s41419-019-2062-7)
Supplement: Supplementary file 3 [file 41419_2019_2062_MOESM3_ESM.docx]

The siRNA and shRNA sequence used in this paper were listed as follows and * means the chosen siRNA or shRNA for further study:

| Name | Sequence |
| --- | --- |
| shSCR | TTCTCCGAACGTGTCACGT |
| shGATA3#1 | GAGAAAGAGTGCCTCAAGTAC |
| shGATA3#2 | GCAGTATCATGAAGCCTAAAC |
| shGATA3#3 | CATCCAGACCAGAAACCGAAA* |
| shDicer#1 | GAGTAATGCTGAAACTGCAACTGAC |
| shDicer#2 | TAAAGTAGCTGGAATGATG* |
| shDicer#3 | GAATATCGATCCTATGTTC |
| shUTX#1 | GCATTTCAGGAGGTGCTTTAT |
| shUTX#2 | GCACTTGCAGCACGAATTAAG |
| shUTX#3 | GCTTCTGGTTAACCACAAACC* |
| siControl | TTCTCCGAACGTGTCACGT |
| siERα | CGAGUAUGAUCCUACCAGAtt |
| siGATA3#1 | CUCUGGAGGAGGAAUGCCAdTdT* |
| siGATA3#2 | CGCAGUACCCGCUGCCGGAdTdT |
| siGATA3#3 | GGGUUAGAGCCCUGCUCGAdTdT |
| siUTX#1 | GCAUUUCAGGAGGUGCUUUdTdT* |
| siUTX#2 | GACAACAAGGCAUUACCUUdTdT |
| siUTX#3 | GUCAAUAGUACCCAUGGUUdTdT |

The primers used in RT-PCR were listed as following:

| Gene | Strand | Sequence |
| --- | --- | --- |
| TNF | F | CCTCTCTCTAATCAGCCCTCTG |
| TNF | R | GAGGACCTGGGAGTAGATGAG |
| SOCS3 | F | CCTGCGCCTCAAGACCTTC |
| SOCS3 | R | GTCACTGCGCTCCAGTAGAA |
| CX3CL1 | F | GCCACAGGCGAAAGCAGTA |
| CX3CL1 | R | GGAGGCACTCGGAAAAGCTC |
| UTX | F | GGACATGCTGTGTCACATCCT |
| UTX | R | CTCCTGTTGGTCTCATTTGGT |
| MAP3K14 | F | CGGAAAGTGGGAGATCCTGAA |
| MAP3K14 | R | GGGCGATGATAGAGATGGCAG |
| CAV1 | F | CATCCCGATGGCACTCATCTG |
| CAV1 | R | TGCACTGAATCTCAATCAGGAAG |
| Dicer | F | GAGCTGTCCTATCAGATCAGGG |
| Dicer | R | ACTTGTTGAGCAACCTGGTTT |
| DUSP2 | F | GGGCTCCTGTCTACGACCA |
| DUSP2 | R | GCAGGTCTGACGAGTGACTG |
| KIT | F | CGTTCTGCTCCTACTGCTTCG |
| KIT | R | CCCACGCGGACTATTAAGTCT |
| L1CAM | F | TGTCATCACGGAACAGTCTCC |
| L1CAM | R | CTGGCAAAGCAGCGGTAGAT |
| RAC1 | F | ATGTCCGTGCAAAGTGGTATC |
| RAC1 | R | CTCGGATCGCTTCGTCAAACA |
| TCF3 | F | ACGAGCGTATGGGCTACCA |
| TCF3 | R | GTTATTGCTTGAGTGATCCGGG |
| SOX2 | F | GCCGAGTGGAAACTTTTGTCG |
| SOX2 | R | GGCAGCGTGTACTTATCCTTCT |
| LAMC1 | F | ACTGCCACTGACATCAGAGTA |
| LAMC1 | R | GCTTGCGTGTCCATTACATTTAC |
| CDK14 | F | TGGGAAGTTGGTAGCTCTGAA |
| CDK14 | R | CCAGGGTGCTTGTCCATGTA |
| PAK1 | F | CAGCCCCTCCGATGAGAAATA |
| PAK1 | R | CAAAACCGACATGAATTGTGTGT |
| GATA3 | F | GCCCCTCATTAAGCCCAAG |
| GATA3 | R | TTGTGGTGGTCTGACAGTTCG |
| GAPDH | F | GGAGCGAGATCCCTCCAAAAT |
| GAPDH | R | GGCTGTTGTCATACTTCTCATGG |
| MMP2 | F | TACAGGATCATTGGCTACACACC |
| MMP2 | R | GGTCACATCGCTCCAGACT |
| MMP9 | F | TGTACCGCTATGGTTACACTCG |
| MMP9 | R | GGCAGGGACAGTTGCTTCT |
| Integrin α5 | F | GGCTTCAACTTAGACGCGGAG |
| Integrin α5 | R | TGGCTGGTATTAGCCTTGGGT |
| Integrin β1 | F | CCTACTTCTGCACGATGTGATG |
| Integrin β1 | R | CCTTTGCTACGGTTGGTTACATT |

The primers used in ChIP assays were listed as following:

| Gene | Strand | Sequence |
| --- | --- | --- |
| UTX | F | AAATGTTACTGTTGTCTAGGGGTTAC |
| UTX | R | TGATTTCAGTGGAGCCTTCTTAC |
| PTEN | F | TGAAATTCAACGGCTATGTGT |
| PTEN | R | GCAACCTGCTATTGTGTCGC |
| ERα | F | ATTTTACAGCCCCTTTGC |
| ERα | R | TCACACTCTTCAGCCCTTC |
| AXIN1 | F | ACACTCCAATGGCCTTGCTC |
| AXIN1 | R | GGACCCTCTGCCTTGATTTCTC |
| Dicer | F | GATTAGCATTTGGGTTGG |
| Dicer | R | TGTCTGAGATCAGGAGGC |
| LIMA1 | F | GGGGAAACCTGTTGAGTA |
| LIMA1 | R | AGAGTATGTATGTGCCTTG |
| FFAR2 | F | CAGACGAACGGAAGTAGGAA |
| FFAR2 | R | CAGTATAAAGAGGCTGTGGC |
| SATB2 | F | TCGGACTTCTTTGGAACTTGGT |
| SATB2 | R | GCACGGGCTTAGAGGCTTG |
| GSN | F | GGGTGGATACCTTGCTTG |
| GSN | R | CAGTCTACTCTTCTATGCCTGA |
| PTCH1 | F | CCCTGAACTTCTTCCTCCTGC |
| PTCH1 | R | CACCTTTATTTCGGCTCCCTC |
| GAPAH | F | TACTAGCGGTTTTACGGGCG |
| GAPDH | R | TCGAACAGGAGGAGCAGAGAGCGA |
